# Vorticity budget analysis of a mesoscale convective vortex during the July 2022 flash flood in Northern Tehran

**DOI:** 10.1038/s41598-026-35778-x

**Published:** 2026-01-13

**Authors:** Nafiseh Pegahfar, Maryam Gharaylou, Omid Alizadeh

**Affiliations:** 1 Atmospheric Sciences Research Center, Iranian National Institute for Oceanography and Atmospheric Science, Tehran, Iran; 2 Institute of Geophysics, University of Tehran, Tehran, Iran; 3 Geography Department, Humboldt-Universität zu Berlin, Berlin, Germany

**Keywords:** Mesoscale convective vortex (MCV), Vorticity budget, Extreme rainfall, Flash flood, Vorticity tendency, ERA5, Climate sciences, Hydrology, Natural hazards

## Abstract

Mesoscale Convective Vortices (MCVs) are influential mid-tropospheric systems capable of initiating or enhancing deep convection, yet their role in extreme precipitation over complex terrain remains insufficiently understood. This study examines the dynamical processes leading to the formation and intensification of an MCV associated with the 27 July 2022 heavy rainfall and deadly flash flood in northern Tehran. Using ERA5 data, GPM precipitation estimates, infrared satellite imagery, and Doppler radar observations, we document the multiscale evolution of the event and diagnose the vorticity budget throughout the troposphere. A coherent positive vorticity emerged south of the flood site in the late afternoon and migrated northward as convection intensified. Vorticity-budget diagnostics reveal that all four terms, horizontal advection, vertical advection, divergence (stretching), and tilting, made non-negligible contributions to the local vorticity tendency. Horizontal advection dominated the early development of the MCV, producing positive vorticity tendencies of order 10⁻⁸ s⁻² in the 700–600 hPa layer roughly three hours before rainfall onset. Divergence and tilting further amplified cyclonic vorticity below ~ 700 hPa, with tilting peaking near 850 hPa as strong vertical shear strengthened horizontal vorticity conversion. In contrast, vertical advection acted persistently as a compensating term, partially offsetting the low-level vorticity growth. The combined effect of these processes was a vertically coherent vortex column, aligned temporally with radar-observed convective organization. The extreme rainfall resulted from the interaction between a warm, humid monsoonal inflow from lower latitudes and a cooler air mass advected from higher latitudes, which enhanced low-level convergence, mesoscale ascent, and vortex stretching. This dynamically driven coupling between synoptic forcing, mesoscale vorticity generation, and complex topography produced an unexpectedly intense convective system. The findings highlight the importance of MCV-related vorticity processes in triggering high-impact precipitation events in mountainous mid-latitude regions and underscore the need for improved representation of these mechanisms in forecasting systems.

## Introduction

Mesoscale Convective Vortices (MCVs) are frequently associated with significant rainfall and play a key role in initiating and sustaining intense precipitation events^1^. The formation of mesoscale vortices within the stratiform region of Mesoscale Convective Systems (MCSs) was first documented in tropical environments^2,3^, though the vortices embedded in midlatitude MCSs tend to be more intense^4^. During the 1980 s and early 1990 s, research on mesoscale vortices relied primarily on conventional observations, including satellite imagery, radar, and soundings, which enabled identification of only the basic structural features of MCVs. For example, Menard and Fritsch (1989) demonstrated that an MCS can generate a mid-level mesoscale vortex during its mature and dissipating stages^5^, while Cotton et al.^6^ documented mid-level positive relative vorticity within an MCS, now recognized as an MCV.

MCVs typically exhibit temporal scales from several hours to multiple days and horizontal dimensions of several hundred kilometers, with vertical extents reaching from the surface to the tropopause^7,8^. As large mesoscale structures, they are capable of organizing moist convection^9^. MCV development generally results from the convergence of synoptic-scale vorticity, with Earth’s rotation providing the dominant contribution^10,11^. Favorable environmental conditions include weak background flow, low vertical wind shear, weak ambient vorticity, and strong horizontal and vertical moisture gradients^10^.

The vorticity budget of an MCV provides critical insight into its formation and evolution. In the absence of friction, vertical vorticity arises from horizontal advection of absolute vorticity, vertical advection of relative vorticity, convergence (stretching) of absolute vorticity, tilting of horizontal vorticity into the vertical by wind shear, and horizontal baroclinicity^12,13^. Numerous studies have analyzed these processes to better understand MCV dynamics and improve rainfall prediction^1,13–17^. For instance, Cram et al.^18^ showed in idealized MCS simulations that tilting and stretching are crucial in MCV formation and intensification. Vertical vorticity originates from the tilting of horizontal vorticity, while stretching primarily enhances vortex strength, though the contributions of each term vary with MCV location, size, and intensity.

Several numerical studies have further clarified MCV vorticity dynamics. Min and Yongguang^19^ investigated an MCV in South China and identified low-tropospheric divergence associated with convection as the dominant source of positive vorticity. Tilting transferred horizontal vorticity into the vertical, partially compensating for upward cyclonic vorticity export. Their results also show that planetary vorticity advection is negligible at low latitudes, with relative vorticity tendencies guiding vortex propagation. Convection-driven processes, including latent heat release and tilted wind shear, governed the southward movement and spin-up of the meso-vortex. Likewise, Wang et al.^16^ demonstrated that mid-level stretching and low-level eddy fluxes were key to MCV development along the Meiyu front. Zhang et al.^20^ further showed that vorticity anomalies help drive diurnal cycles of long-lived MCVs, highlighting the roles of enhanced vorticity from the Sichuan Basin, vertical decoupling in the afternoon, low-level jets and radiative processes at night, and convergence-driven intensification prior to offshore movement.

Recent work has extended these findings to extreme rainfall events. Fu et al.^17^ analyzed the vorticity budget during the catastrophic Henan July 2021 rainfall and found convection-driven vortex stretching and upward cyclonic vorticity transport to be central to MCV formation and maintenance. Outward horizontal advection dominated the dissipation stage following decoupling from the parent MCS. Ahmadloo et al.^21^ demonstrated that simulations of Iranian MCSs can realistically represent precipitation patterns, vertical structure, moist layers, and rear-inflow jets. Wu et al.^22^ examined a dual-core Southwest China vortex and highlighted major contributions from stretching and vertical advection, along with significant roles of latent heating and topography. Reanalysis-based studies also underscore the importance of stretching, divergence, and tilting in MCV development, as shown in analyses of the 28 May 1985 event^23^ and MCV activity in East China^1^.

Iran’s highly variable topography and climate, from deserts to high mountain ranges and coastal plains^24^, make the country especially vulnerable to extreme weather. Heavy rainfall^25,26^, floods^27^, and extreme temperatures^28^ routinely cause major socioeconomic impacts. Complex terrain interacts with synoptic and mesoscale circulation patterns to shape vortex development and propagation. Recent analyses of the widespread July 2022 flooding indicate that tropical monsoon inflow, midlatitude disturbances, enhanced jet stream activity, and synoptic-scale convergence jointly contributed to extreme precipitation^29^. Similar mechanisms have recently been documented over the Arabian Peninsula, where an extreme MCS-driven rainfall event in April 2024 resulted from the rare coupling of intense low-level moisture transport via the Somali low-level jet and a strong mid-tropospheric cold-core vortex, highlighting the importance of multiscale interactions in producing extreme precipitation in arid regions^30^. These conditions may also favor the development and intensification of MCSs and their embedded vortices. Studying the vorticity budget of Iranian MCVs is thus essential for improving understanding of local atmospheric processes and enhancing forecast accuracy and early-warning capability.

Although prior studies have explored some MCS characteristics in Iran^21^, MCVs have received relatively little attention. This gap limits our understanding of their structure, interaction with larger-scale systems, and role in producing hazardous weather. The importance of this knowledge is underscored by the severe monsoonal rainfall and flooding that struck Iran in summer 2022, resulting in 108 fatalities, impacts across 24 provinces, and an estimated USD 3.3 billion in losses^31^. Among the worst-affected locations was Imamzadeh Davood in northern Tehran, where a sudden flash flood on 27 July produced destructive mudflows and 22 deaths^29^. Because these extreme rainfall events may be linked to MCS–MCV interactions, the present study examines the distribution of relative vorticity tendency within the mesoscale vortex associated with the late-July 2022 Tehran event. Our goal is to clarify the dynamical characteristics of the MCV and its role in producing the observed heavy rainfall.

## Data and methodology

### Data sources

This study utilized the European Centre for Medium-Range Weather Forecasts 5th Generation Reanalysis (ERA5)^32^, which provides hourly data at a horizontal resolution of 0.25° × 0.25°. The ERA5 variables used in this study include zonal wind (u), meridional wind (v), vertical pressure velocity (ω), and pressure fields from 1000 to 100 hPa. These fields were employed to calculate the vertical vorticity budget terms throughout the troposphere.

To complement ERA5, 24-hour accumulated rainfall data from the late-run Global Precipitation Measurement (GPM) satellite^33^ were used, providing a horizontal resolution of 0.1° (~ 10 km) for the study area. While ERA5 offers high temporal resolution and global coverage, it may not fully resolve mesoscale or convective-scale processes, potentially underestimating rapid vorticity changes. ERA5 is generated by combining numerical models with data assimilation of available observations, including satellite, radar, and in-situ measurements. Despite this, biases can occur, particularly in observation-sparse regions like Iran, which may propagate into derived quantities such as vorticity.

All times in this study are given in Coordinated Universal Time (UTC) unless otherwise stated; local time (LT) in Tehran in July 2022 was UTC + 4:30.

### Vorticity budget calculation

To investigate the physical processes behind vorticity generation, all terms of the vertical vorticity equation were evaluated using three-dimensional wind fields. Ignoring friction and subgrid-scale effects, the equation in pressure coordinates can be expressed as^34^:

$$\:{\left(\frac{\partial\:{\xi\:}_{p}}{\partial\:t}\right)}_{p}=-\left[u{\left(\frac{\partial\:\left({\xi\:}_{p}+f\right)}{\partial\:x}\right)}_{p}+v{\left(\frac{\partial\:\left({\xi\:}_{p}+f\right)}{\partial\:y}\right)}_{p}\right]-\omega\:{\left(\frac{\partial\:{\xi\:}_{p}}{\partial\:p}\right)}_{p}-\left({\xi\:}_{p}+f\right)\left[{\left(\frac{\partial\:u}{\partial\:x}\right)}_{p}+{\left(\frac{\partial\:v}{\partial\:y}\right)}_{p}\right]+\left[{\left(\frac{\partial\:\omega\:}{\partial\:y}\right)}_{p}\frac{\partial\:u}{\partial\:p}-{\left(\frac{\partial\:\omega\:}{\partial\:x}\right)}_{p}\frac{\partial\:v}{\partial\:p}\right]\:\:\:\:\:\:\:\:\:\:\:\:\:\:\:\:\:\:\:\:\:\:\:\:\left(1\right)$$

where $\:f$ is the Coriolis parameter, $\:\omega\:=\partial\:p/\partial\:t$ is the vertical velocity, and $\:{{\xi\:}_{p}=\left(\frac{\partial\:v}{\partial\:x}\right)}_{p}-{\left(\frac{\partial\:u}{\partial\:y}\right)}_{p}$is the vertical component of relative vorticity, with $\:u$ and $\:v$ representing the two horizontal wind components. The subscript $\:p$ indicates pressure coordinates and is omitted for convenience in the following discussion.

### Vorticity tendency components

The terms on the right-hand side represent the main contributors to the vorticity tendency:

- Horizontal advection: $$\:{\xi\:}_{hadv}=-\left(u\frac{\partial\:\left(\xi\:+f\right)}{\partial\:x}+v\frac{\partial\:\left(\xi\:+f\right)}{\partial\:y}\right)$$

Represents the advection of absolute vorticity by the non-uniform horizontal flow.

- Vertical advection: $$\:{\xi\:}_{vadv}=-\omega\:\left(\frac{\partial\:\xi\:}{\partial\:p}\right)$$

Represents the vertical transport of relative vorticity by vertical motion.

- Divergence (stretching): $$\:{\xi\:}_{div}=-\left(\xi\:+f\right)\left(\frac{\partial\:u}{\partial\:x}+\frac{\partial\:v}{\partial\:y}\right)$$

Indicates amplification (or reduction) of vertical vorticity due to horizontal convergence (or divergence).

- Tilting: $$\:{\xi\:}_{tilt}=-\left(\frac{\partial\:\omega\:}{\partial\:x}\frac{\partial\:v}{\partial\:p}-\frac{\partial\:\omega\:}{\partial\:y}\frac{\partial\:u}{\partial\:p}\right)$$

Represents the generation of vertical vorticity from the tilting of horizontal vorticity by non-uniform vertical motion.

The local tendency of relative vorticity represents the local variation of relative vorticity and can be expressed as:

$$\:{\xi\:}_{ten}=\frac{\partial\:{\xi\:}_{p}}{\partial\:t}={\xi\:}_{hadv}+{\xi\:}_{vadv}+{\xi\:}_{div}+{\xi\:}_{tilt}$$

### Computation approach

Using ERA5 data, all terms on the right-hand side were computed at each grid point and isobaric surface. The sum of these terms provides the local relative vorticity tendency ($\:{\xi\:}_{ten}$) at each location. Domain averages over the vortex area were then calculated to assess the overall contributions of each vorticity budget term to the development of mesoscale convective vortices (MCVs) during the event.

## Study area and climatology

On 27–28 July 2022, a heavy rainfall event affected multiple regions of Iran, causing widespread flooding and severe weather, particularly in northern Tehran. Tehran is situated in the southern foothills of the Alborz Mountains, between the mountains and the northern dry plains (35°14′–36°17′ N, 50°14′–53°6′ E), at an elevation of 1000–1800 m. The Alborz Mountains block Caspian rain-bearing winds, resulting in cooler and wetter conditions in the northern hilly areas compared to the flat southern plains^35,36^. The surrounding topography also strongly influences local wind patterns: during the night, northwesterly to westerly winds from the mountains toward the plains dominate, particularly in the western half of Tehran, while during the daytime prevailing winds are southwesterly, from the plains toward the mountains^36^.

The climate of Tehran is semi-arid, with hot, dry summers and cool, semi-dry winters, during which Rossby-forced advection of Mediterranean air occasionally produces weak to moderate, and occasionally heavy, precipitation events^35^. Long-term observations at Mehrabad Airport (1951–2013) indicate that summer (June–August) and winter (December–February) daily mean temperatures are 29.1 °C and 5.1 °C, respectively, representing the west-central lowlands of the city^35^. Annual precipitation in these lowlands averages 233.4 mm, with the majority falling between November and April, and minimal rainfall occurring from June to September. Monthly precipitation peaks in March (39.1 mm) and is lowest in September (1.1 mm)^35^. In contrast, the northern highlands receive an average annual precipitation of 422.2 mm and have a mean annual temperature of 15.5°C^35^.

Overall, the southern plains exhibit a semi-arid steppe climate, while the northern mountainous areas have an alpine climate^37^. The combination of complex topography and north–south climatic gradients contributes to frequent low winds, poor ventilation, and the region’s vulnerability to flash floods and extreme heatwaves^38^.

## Results

### Synoptic conditions

**Fig. 1:**
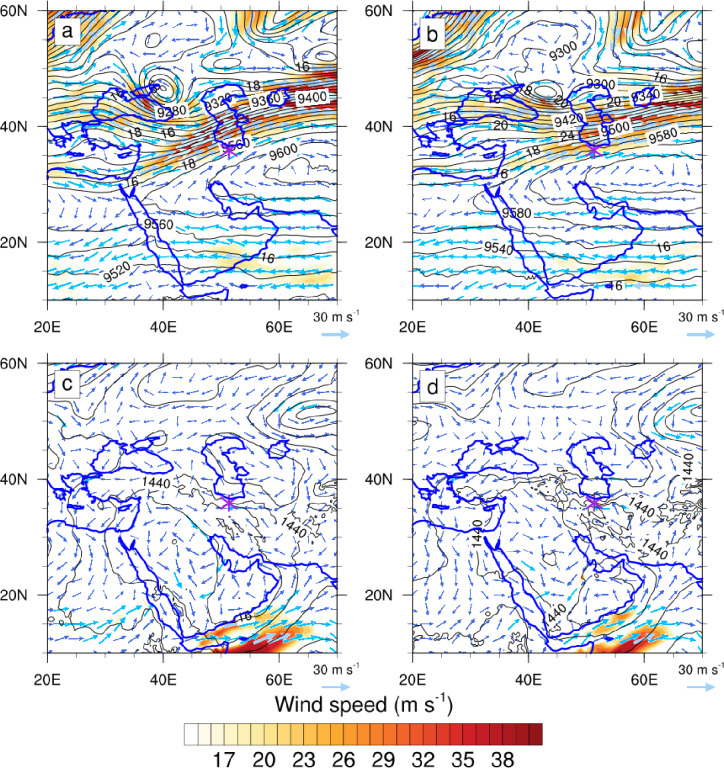
Latitude–longitude distribution of meteorological fields during 27 July 2022. (**a**, **b**) Geopotential height (contours, in gpm) and wind speed (vectors and shaded areas, in m s⁻¹) at 300 hPa; (**c**, **d**) geopotential height (contours, in gpm) and wind speed (vectors and shaded areas, in m s⁻¹) at 850 hPa. Panels a, c (**b**, **d**) correspond to 06:00 UTC (18:00 UTC). The purple star indicates the location of the Imamzadeh Davood flood event.

At 06:00 UTC (10:30 LT) on 27 July, a branch of the polar jet stream deviated southward from its typical seasonal position, converging with the northward-shifted subtropical jet over northern Iran (Fig. 1a–b). Such jet interactions often trigger intense rainfall in spring and autumn^38^. Maximum wind speeds reached 25 m/s in the jet stream region. At the surface, a westward branch of high pressure over eastern Iran produced winds up to 20 m/s (Fig. 1c–d), and the convergence of these westerlies with flows from the Black Sea and eastern Mediterranean enhanced low-level convergence. Divergence in the entrance region of the upper-level jet induced vertical motion, strengthening 850 hPa winds and promoting upward motion in the region.

**Fig. 2:**
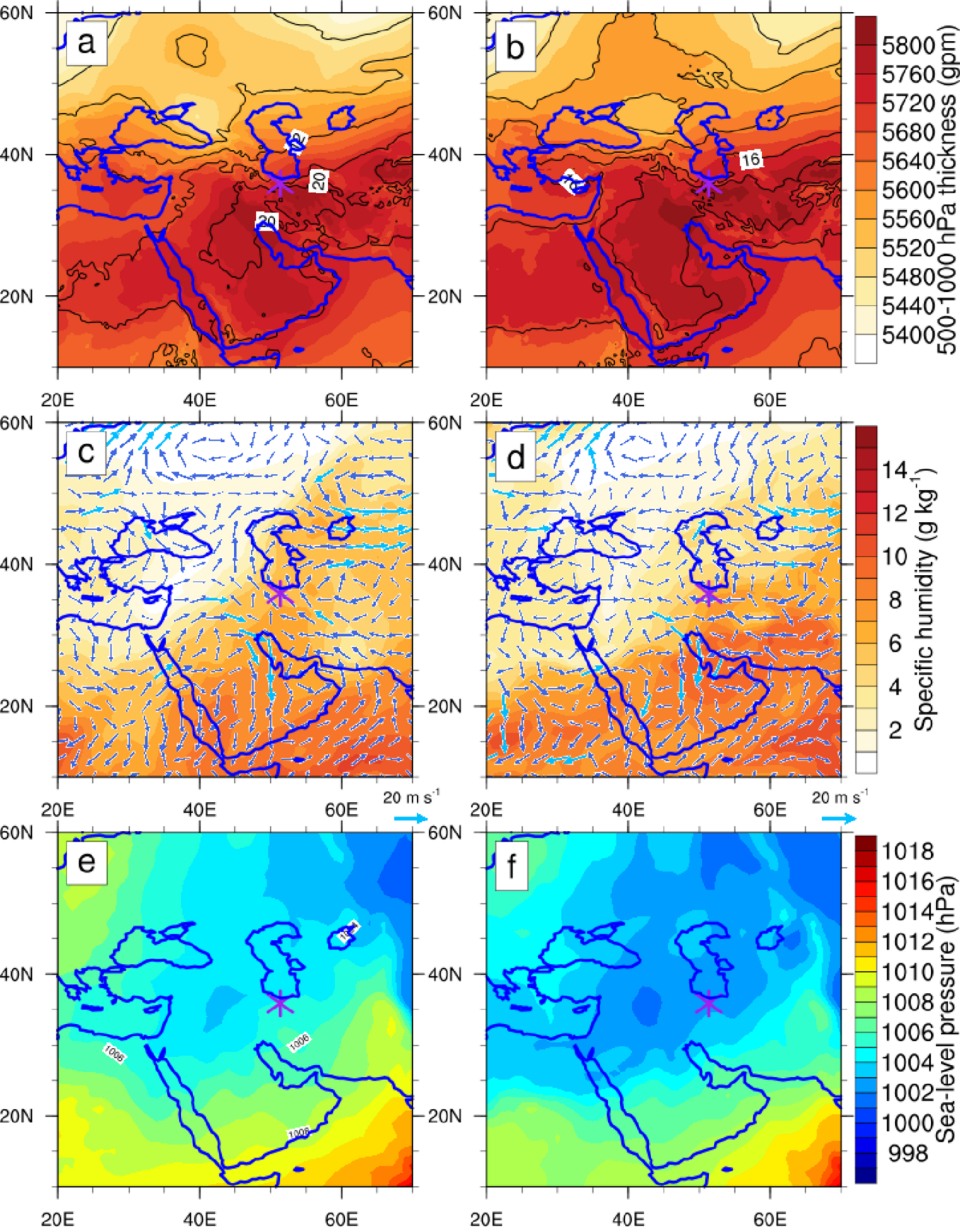
Latitude–longitude distribution of meteorological fields on 27 July 2022. (**a**, **b**) 500–1000 hPa thickness (shaded, in gpm) and temperature (contours, in °C); (**c**, **d**) specific humidity (shaded, in g kg⁻¹) and horizontal wind vectors at 700 hPa (m s⁻¹); (**e**, **f**) sea-level pressure (contours, in hPa). The left (right) columns correspond to 06:00 UTC (18:00 UTC). The purple star indicates the location of the Imamzadeh Davood flood event.

The 500–1000 hPa thickness fields indicate cold advection from higher latitudes and warm advection from lower latitudes (Fig. 2a–b). At 06:00 UTC, warmer, thicker air advected from the south and east toward the region, while cooler, thinner air moved northward and westward (Fig. 2a). By 18:00 UTC, the warm advection zone shifted slightly east-southeast, with elevated thickness and warmer isotherms persisting near the study area (Fig. 2b).

Moisture transport from the Indian Ocean via monsoon flows reached up to 700 hPa, with specific humidity exceeding 10 g/kg (Fig. 2c–d). A surface low-pressure system over central Iran enhanced vertical motion, facilitating the northward movement of humid air from southern regions (Fig. 2e–f). Overall, sustained moisture influx combined with the surface low-pressure system was the primary driver of the flood, with upper-tropospheric influences from the 300 hPa jet playing a secondary role.

### Rainfall and convective activity

**Fig. 3:**
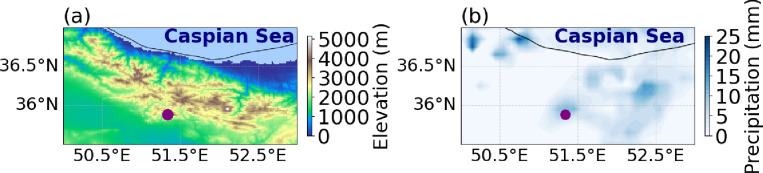
(**a**) Latitude–longitude distribution of elevation above sea level (m) based on ETOPO data (https://gadm.org/). (**b**) 24-hour accumulated rainfall (mm) from GPM data (https://giovanni.gsfc.nasa.gov/) starting at 00:00 UTC on 27 July 2022. The purple dots indicate the location of the Imamzadeh Davood flood event.

The study area exhibits complex topography, with elevation increasing from the southern plains to the Alborz Mountains (Fig. 3a). On 27 July, heavy rainfall was observed, with 24-hour accumulated precipitation derived from GPM satellite data concentrated in the northern mountainous areas (Fig. 3b).

**Fig. 4:**
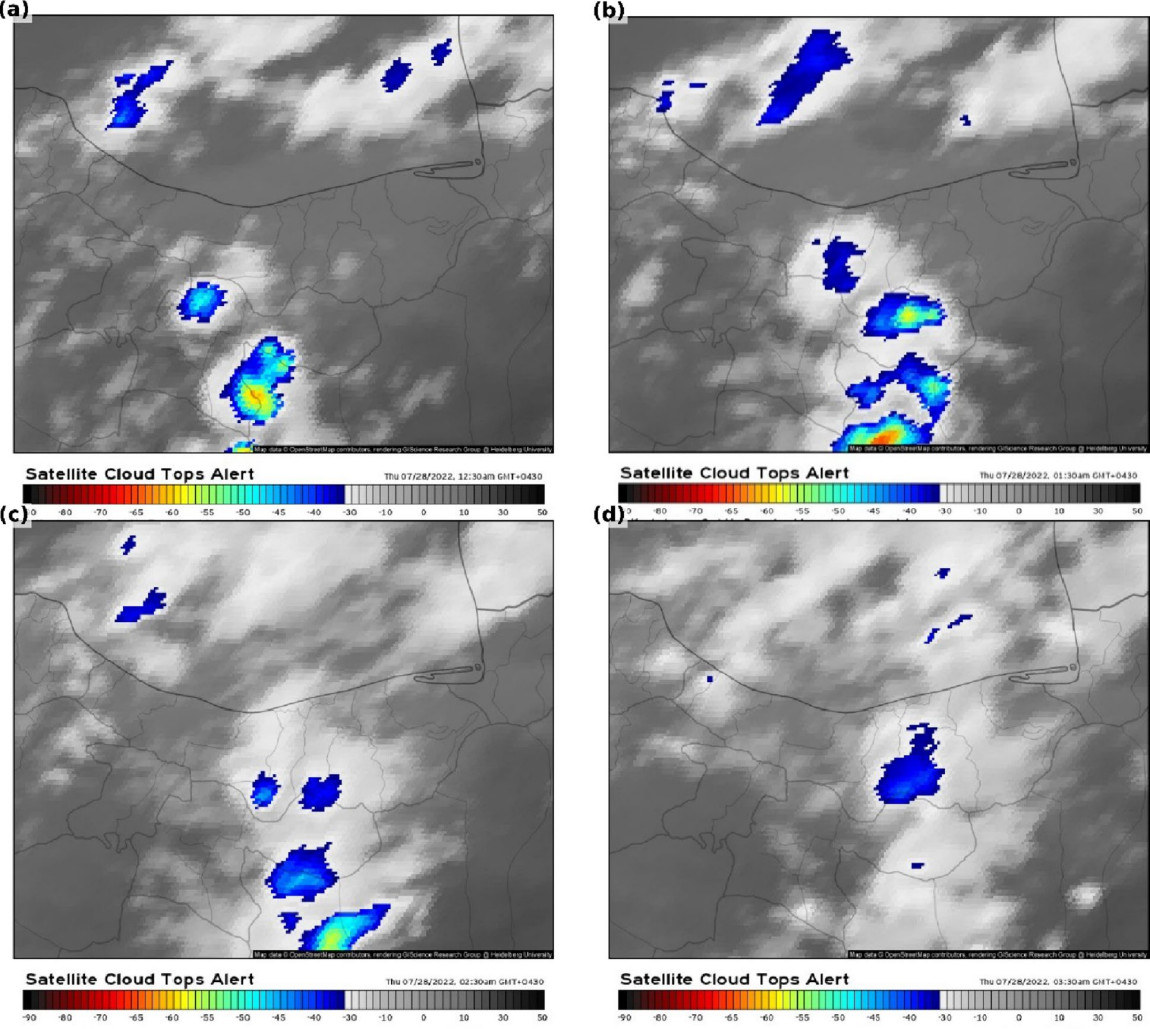
Infrared satellite images from EUMETSAT showing cloud-top temperatures at (**a**) 20:00, (**b**) 21:00, (**c**) 22:00, and (**d**) 23:00 UTC on 27 July 2022. The images were accessed via Meteologix (https://meteologix.com/ir/satellite/top-alert-15 min.html). © Meteologix/Kachelmann Group.

**Fig. 5:**
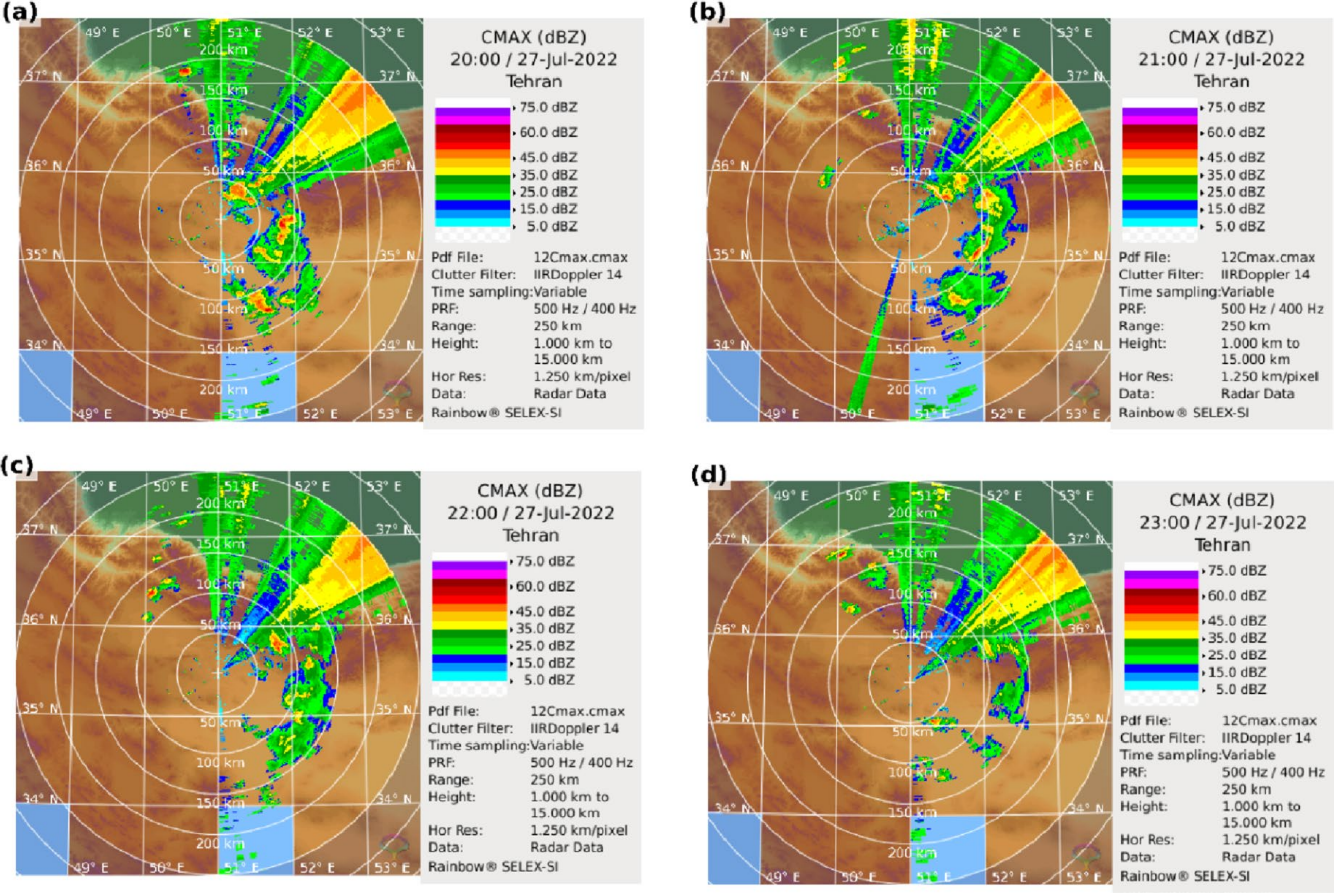
Doppler radar reflectivity (dBZ) observed over Tehran at (**a**) 20:00, (**b**) 21:00, (**c**) 22:00, and (**d**) 23:00 UTC on 27 July 2022, illustrating the evolution of convective activity.

Cloud-top temperatures between 20:00 and 23:00 UTC ranged from − 45 °C to − 65 °C, indicating strong convective activity. Temperatures below − 50 °C confirmed the presence of intense MCSs^4,39^ driving extreme rainfall (Fig. 4). Doppler radar observations captured the evolution of the convective storm, with widespread precipitation between 34°–36°N and 51°–52°E during 20:00–21:00 UTC. A line-shaped reflectivity feature reached 40 dBZ at 20:00 UTC (Fig. 5a), and the system gradually dissipated by 23:00 UTC (Fig. 5a–d), confirming the timing, spatial extent, and intensity of the MCS responsible for the flooding.

### MCS dynamics and potential vorticity

**Fig. 6:**
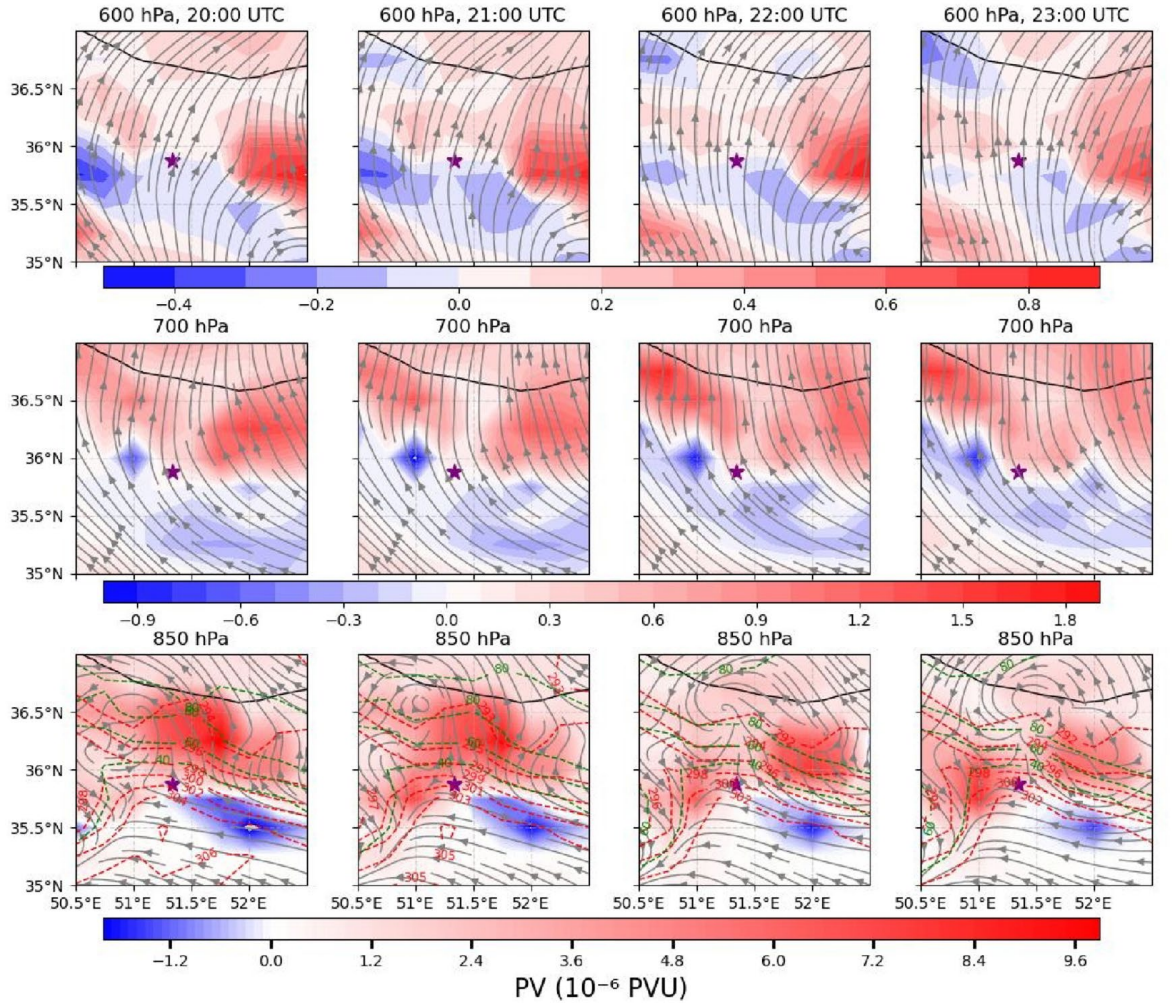
Horizontal distributions of potential vorticity (PV; ×10⁻⁶ PVU, shaded) and wind speed (streamlines; m s⁻¹) at 600 hPa (top row), 700 hPa (middle row), and 850 hPa (bottom row) at 20:00, 21:00, 22:00, and 23:00 UTC on 27 July 2022 (left to right). At 850 hPa, temperature (K; red dashed contours) and relative humidity (%; green dashed contours) are overlaid. The star indicates the location of the Imamzadeh Davood flood event.

To examine the development of the mesoscale convective vortex (MCV) responsible for the severe rainfall, potential vorticity (PV) was analyzed alongside other meteorological variables to assess mid- to lower-tropospheric structure. Vertical profiles of PV at 600, 700, and 850 hPa were used to characterize the vortex evolution (Fig. 6). At 600 hPa, PV primarily reflects mid- to upper-level cyclonic vorticity, whereas at 700 and 850 hPa, low-level circulation and outflow boundaries from convective systems become more pronounced.

Between 20:00 and 23:00 UTC on 27 July, southerly to southeasterly flow dominated the region. Negative PV prevailed at 600 hPa, while positive PV cells in higher latitudes intensified to 1 PVU. Negative PV centers in the southeast and southwest weakened from − 0.5 to − 0.2 PVU. At 700 hPa, both positive and negative PV centers strengthened, reaching 2 PVU and − 1 PVU, respectively. At 850 hPa, PV centers intensified further (10 PVU and − 2 PVU) and underwent notable spatial reconfiguration, indicating cyclogenesis primarily originating in the lower troposphere. These observations suggest that near-surface and boundary-layer processes were the main drivers of the developing disturbance, with mid-level cyclonic activity playing a secondary role.

Lower-tropospheric temperature and relative humidity fields highlight warm advection in the eastern domain, cold advection in the southwest, dry air advected from the southeast, and moist air from the west (Fig. 6, lower panels). These mesoscale features are consistent with synoptic-scale patterns in Fig. 2, where 500–1000 hPa thickness and temperature fields indicate warm and cold advection, and 700 hPa wind vectors and specific humidity depict the distribution of moist and dry advection.

### Temporal and vertical evolution of PV dipoles

**Fig. 7:**
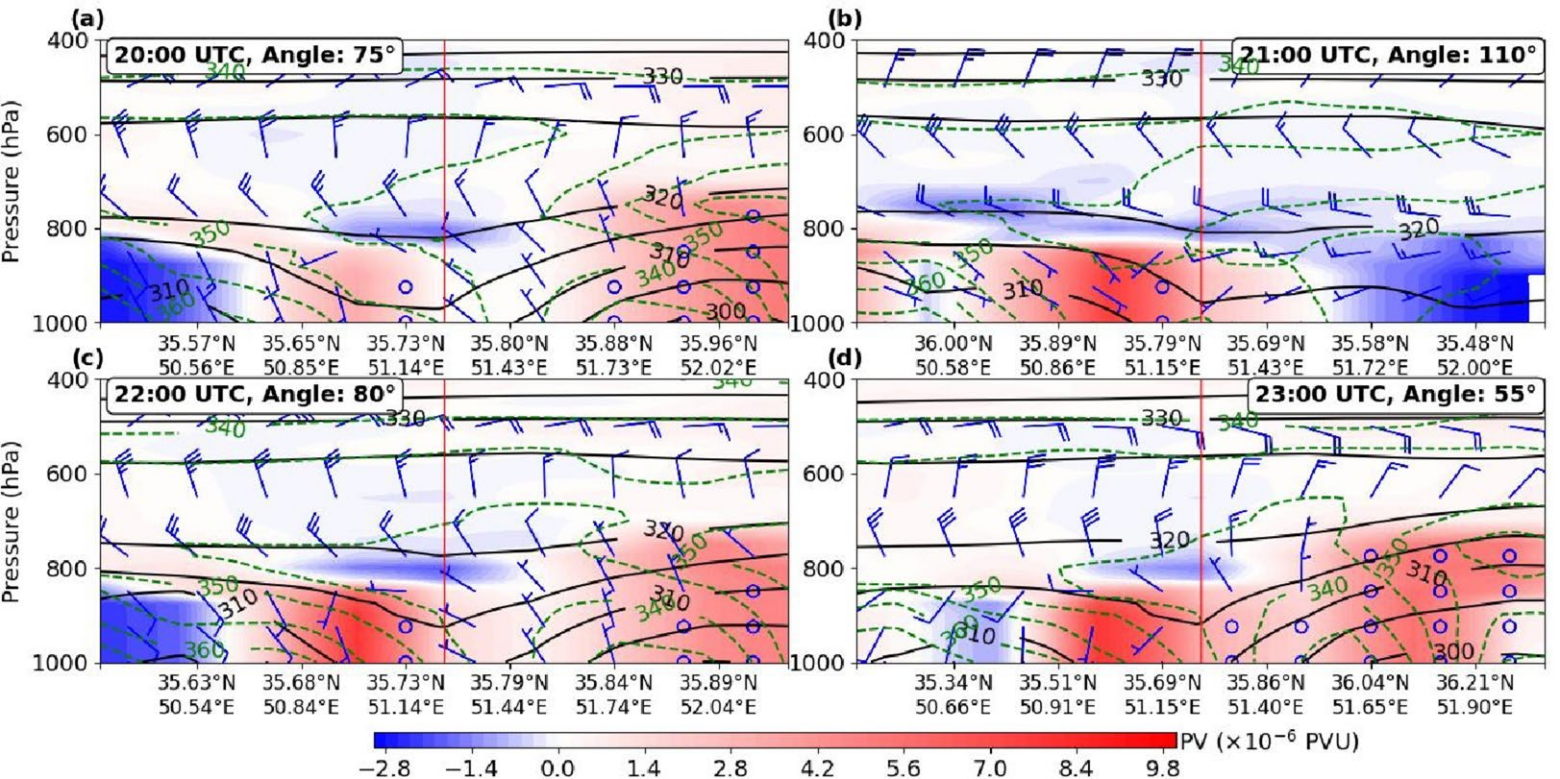
Vertical cross-section of potential vorticity (PV, × 10⁻⁶ PVU, shaded), equivalent potential temperature (θₑ, K, green dashed contours), and potential temperature (θ, K, black solid contours) at (**a**) 20:00, (**b**) 21:00, (**c**) 22:00, and (**d**) 23:00 UTC. The cross-sections are oriented along wind directions of 75°, 110°, 80°, and 55° (wind rose) for each respective time. The vertical red line indicates the location of the nearest grid point to the study area. Horizontal axes show the latitude and longitude of the section. Wind barbs are plotted with half-barbs representing 2 m/s and full barbs representing 4 m/s.

During the event, PV dipoles exhibited significant temporal and vertical variability. Two positive PV centers in the lower atmosphere deepened upward to around 800 hPa in the west and 700 hPa in the east (Fig. 7a), while two negative PV centers were observed, one in the lower troposphere (from the surface to near 800 hPa in the west, Fig. 7a) and another near 800 hPa (Fig. 7a). Between 20:00 and 22:00 UTC, negative PV centers intensified to approximately − 3 PVU. The western positive-PV pole was located between the lower-level negative PV center and an upper-level negative PV feature, which in some areas folded toward the surface (Fig. 7b). At the storm’s onset, the negative PV center extended below 400 hPa; however, the absence of significant mid-tropospheric PV indicates no tropopause folding, contrasting with observations reported by gh et al.^40^. Equivalent potential temperature contours at 22:00–23:00 UTC (Fig. 7c and d) reveal folding patterns that indicate the convergence and interaction of two air masses with distinct thermal characteristics, facilitating the formation of two separate PV cells.

### Vortex evolution and connection to rainfall

**Fig. 8:**
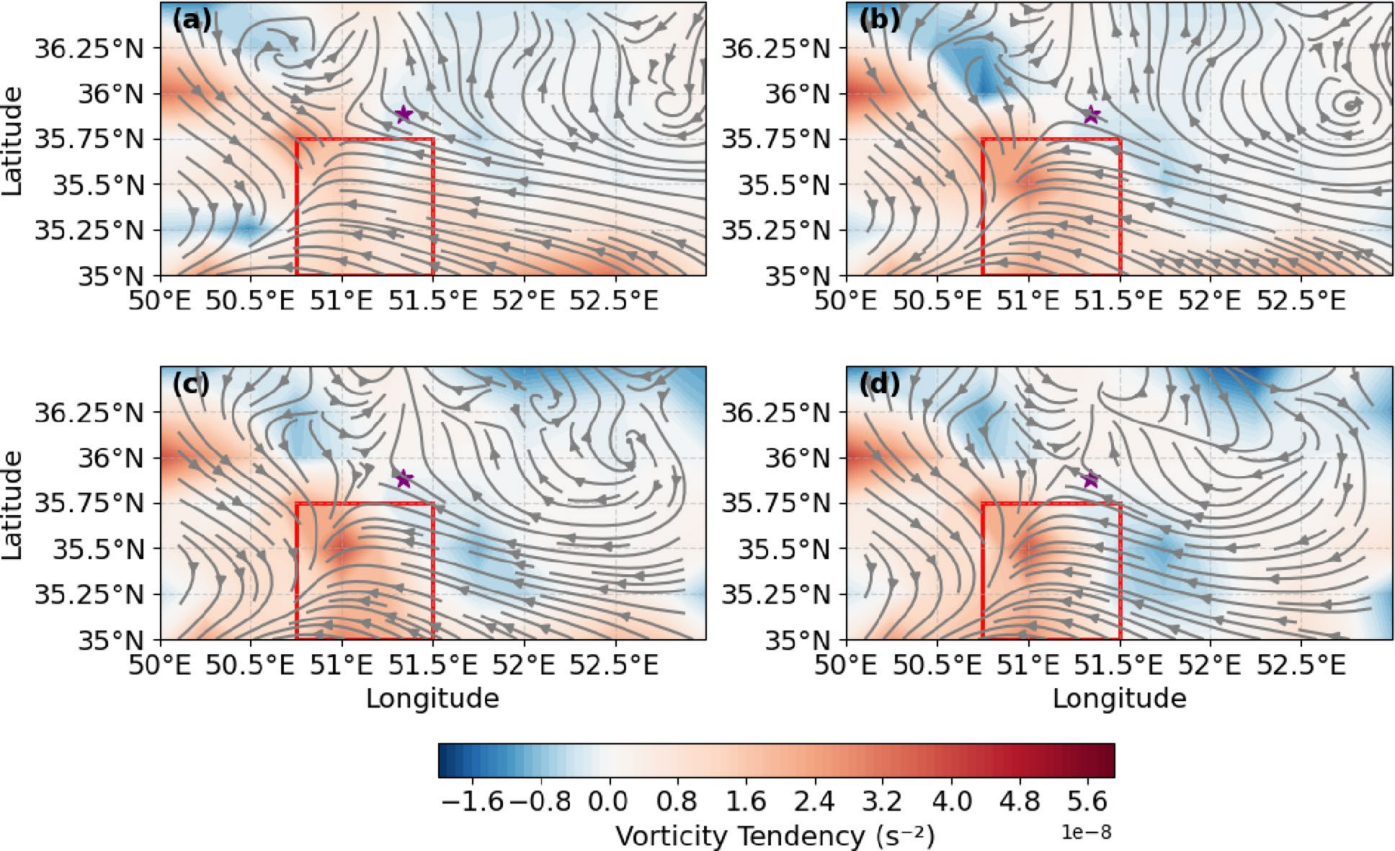
850 hPa wind vectors (m s^− 1^) and vorticity tendency (× 10⁻⁸ s⁻², color-shaded) on 27 July 2022 at (**a**) 20:00 UTC, (**b**) 21:00 UTC, (**c**) 22:00 UTC, and (**d**) 23:00 UTC. The location of Imamzadeh Davood is marked with a purple star, and the red rectangle (34.9°–35.7°N, 51.2°–51.8°E) highlights a region of enhanced positive vorticity tendency.

The evolution of the vortex center further illustrates the connection between PV structures and surface rainfall. The vortex initially formed southwest of 51.3°E, 35.8°N, corresponding to the location of the Imamzadeh Davood flood event (Fig. 8a). This positive vorticity center coincided with areas of strong radar reflectivity (> 55 dBZ) observed earlier (Fig. 5a–b). By 21:00 UTC, the MCV shifted slowly eastward, with localized increases in precipitation accumulation (Fig. 8b). Around 22:00 UTC, the vortex intensified, producing extreme rainfall in the flood-affected region (Fig. 8c). By 23:00 UTC, both the reflectivity and positive vorticity centers began to weaken, indicating the dissipation phase of the convective system (Figs. 5d and 8).

**Fig. 9:**
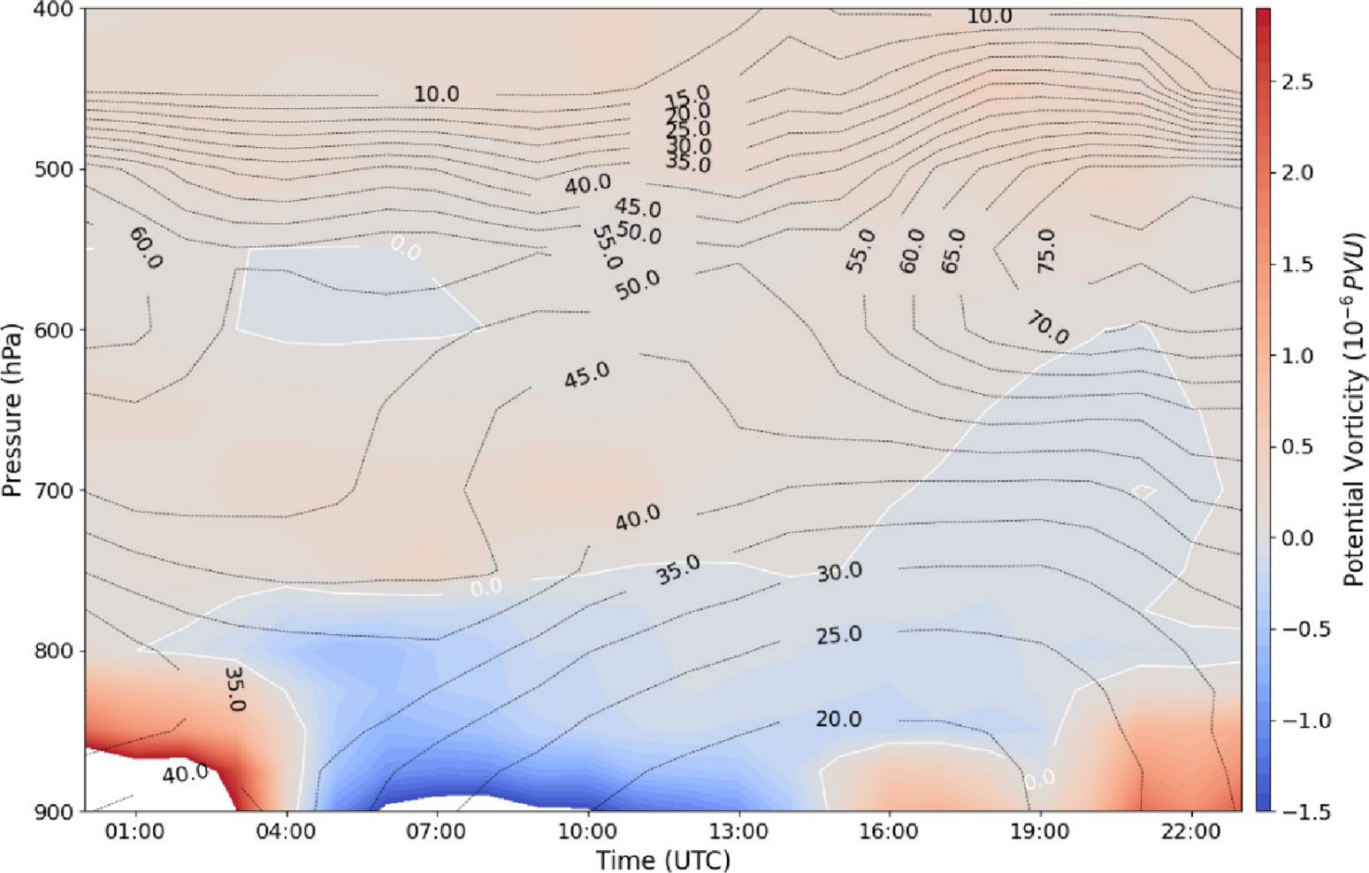
Time–height cross section of potential vorticity (PV, shaded; PVU) and relative humidity (RH, black contours; %) on 27 July 2022. The white line denotes the zero-PV contour. All fields are averaged over the region outlined by the red rectangle in Fig. 8.

By 15:00 UTC on 27 July, negative PV values near the surface had diminished, and a positive PV center began developing at low levels. This center intensified over time, reaching maximum strength by 21:00 UTC and extending upward to approximately 800 hPa. As the event approached the period of heavy precipitation, the positive PV center continued to deepen vertically, while the vertical extent of negative PV in the mid-troposphere diminished (Fig. 9), indicating the strengthening of cyclonic circulation in the lower troposphere and its growing influence on surface rainfall.

### Low-level circulation and pre-flood conditions

**Fig. 10:**
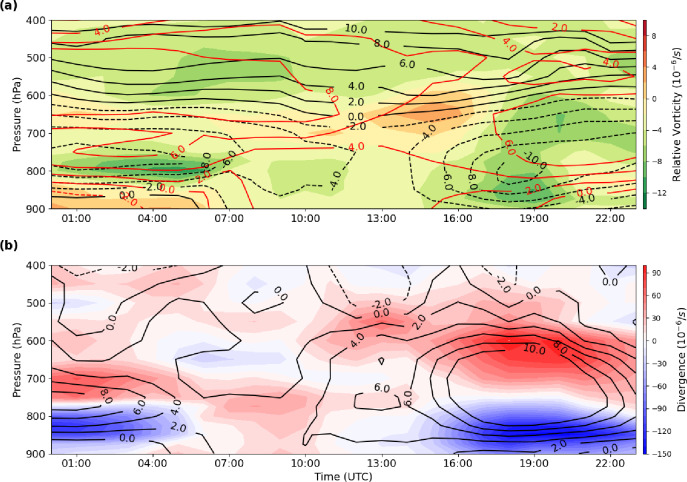
Time–height cross sections on 27 July 2022 of (**a**) relative vorticity (shaded; 10⁻⁶ s⁻¹) and horizontal wind components (u, dashed black lines; v, solid red lines; m s⁻¹), and (**b**) divergence (10⁻⁶ s⁻¹) and vertical velocity (cm s⁻¹). All fields are averaged over the region outlined by the red rectangle in Fig. 8.

Early on 27 July, negative vorticity was present in the atmospheric column south of the flood area, with maximum intensity below 800 hPa (Fig. 10a). Strong easterly zonal winds at 800 hPa and northerly meridional winds at 700 hPa indicate that low-level easterly flow transported warm, humid monsoonal air into the region, while northerly winds advected cooler air from higher latitudes into the mid-troposphere. The resulting convergence of moisture in the lower troposphere coincided with divergence at mid-levels, while upward motion persisted throughout the troposphere (Fig. 10b), creating favorable conditions for deep convection and subsequent MCV formation.

### Vorticity budget and vertical profiles

**Fig. 11:**
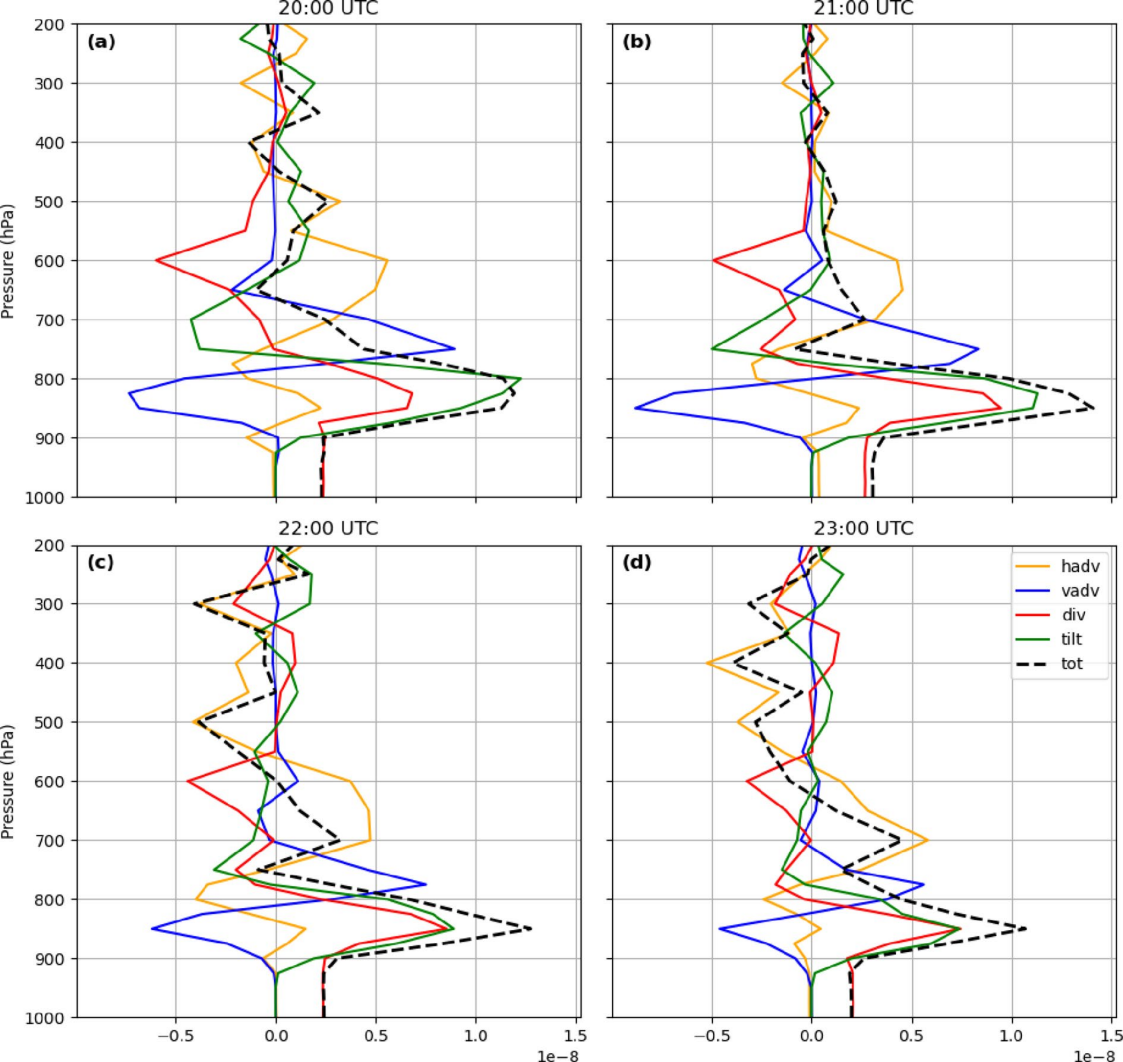
Vertical profiles of the mean values of all terms in Eq. 1, averaged over the red area shown in Fig. 8, at (**a**) 20:00 UTC, (**b**) 21:00 UTC, (**c**) 22:00 UTC, and (**d**) 23:00 UTC on 27 July 2022.

**Fig. 12:**
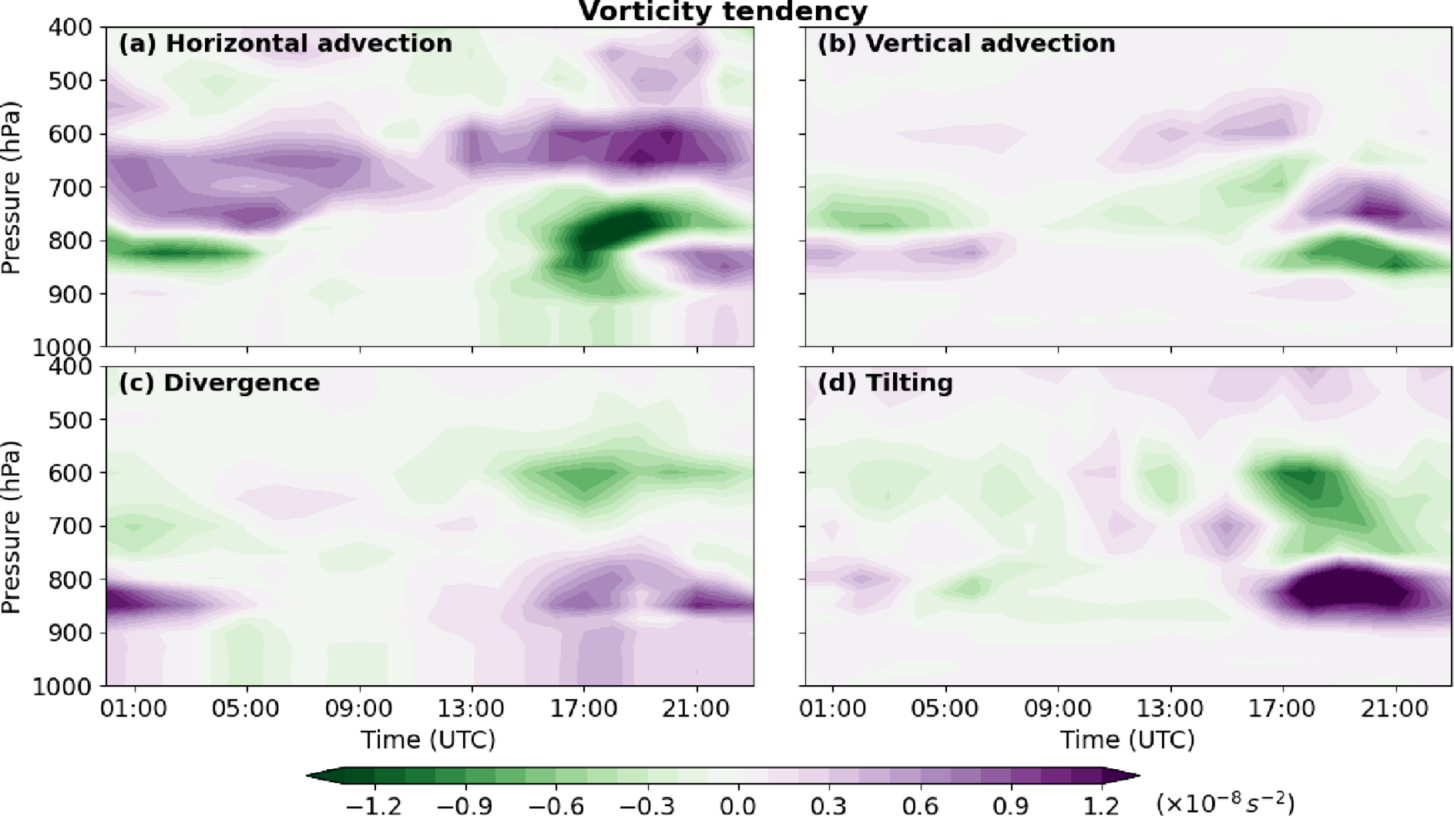
Time evolution (UTC) of the vertical profiles of the vorticity-tendency terms: (**a**) horizontal advection, (**b**) vertical advection, (**c**) divergence, and (**d**) tilting, averaged over the red square shown in Fig. 6.

The vertical profiles of domain-averaged vorticity budget terms (horizontal advection, vertical advection, divergence, and tilting) reveal their evolving contributions throughout the troposphere (Figs. 11 and 12). At 850 hPa, vertical advection ($\:{\xi\:}_{vadv}$) opposed the other three terms, with a magnitude of ~ − 0.5 × 10⁻⁸ s⁻², while horizontal advection, divergence, and tilting were positive (> − 0.5 × 10⁻⁸ s⁻²). Tilting peaked near 1.25 × 10⁻⁸ s⁻² at 20:00 UTC, whereas total vorticity dominated at 21:00 UTC (~ 1.4 × 10⁻⁸ s⁻²). These magnitudes gradually decreased over time, and by 22:00–23:00 UTC, the vertical advection term became positive (~ 0.55 × 10⁻⁸ s⁻²) at 800 hPa, while the horizontal term reached to its minimum value in the lower troposphere (~ − 0.4 × 10⁻⁸ s⁻² at 22:00 UTC and ~ − 0.25 × 10⁻⁸ s⁻² at 23:00 UTC).

The vertical profiles of vorticity tendency terms from 1000 to 200 hPa further illustrate the evolving role of each mechanism in the development of the MCV (Fig. 12). Horizontal advection reached positive values of up to 1.2 × 10⁻⁸ s⁻² around 700–600 hPa in the evening, consistent with meridional wind patterns in the lower troposphere (Fig. 10a). Several hours prior to the flood, a negative center of horizontal advection appeared at lower levels (~ − 1.2 × 10⁻⁸ s⁻²), corresponding to intensified easterly zonal winds that transported warm, humid monsoonal air into the region. Vertical advection became significant during the pre-flood period, forming a dipole near 800 hPa with a positive center aloft and a negative center below (Figs. 10b and 12b), reflecting compensating vertical transport. Divergence and tilting also exhibited dipole structures, with positive centers in the lower troposphere and negative centers aloft, reaching magnitudes of 1–1.1 × 10⁻⁸ s⁻².

The profiles indicate that all four vorticity tendency terms contributed substantially to the development and intensification of the MCV. Horizontal advection, divergence, and tilting primarily enhanced cyclonic vorticity, while vertical advection consistently acted as a compensating force. Positive vorticity extended through most of the atmospheric column, whereas convergence of absolute vorticity was largely confined below ~ 700 hPa, coinciding with maximum horizontal and vertical winds. These results highlight the critical role of low-level processes, modulated by mesoscale circulation and vertical wind shear, in producing the observed extreme rainfall.

## Conclusions

This study investigated a heavy rainfall event that caused flooding in northern Tehran on 27 July 2022. Two primary weather systems influenced the region during this event. The first system, originating from lower latitudes, transported warm and humid air masses generated by monsoon activity over southern waters. These air masses entered the MCV that developed south of the study area. The second system, originating from higher latitudes, carried cold air into the southwestern portion of the domain. The interaction of these distinct air masses south of the flood location played a key role in triggering and intensifying the MCV. The evolution of this MCV occurred over a three-hour period starting at 21:00 UTC on 27 July 2022. This timing aligns with previous studies showing that MCVs predominantly form during late afternoon to early morning hours, consistent with observations reported in Asia and elsewhere^41,42^. Analysis of vertical profiles of meteorological parameters indicated that the MCV initially developed near the surface and did not extend significantly into higher altitudes, supporting earlier findings that MCVs in Asia tend to form at lower altitudes compared to their counterparts in the United States^1^. Moreover, the MCV originated within the convective precipitation zone of an MCS, consistent with prior research focused on Asian cases.

The vorticity budget, including horizontal and vertical advection, divergence, and tilting, was analyzed to characterize the dynamics of the MCV. Both positive and negative vorticity tendencies were evaluated. The results showed that all four terms contributed to the vorticity tendency with similar magnitudes, with vertical advection acting as a compensating force against the other three terms. These findings contrast with the study by Sun et al.^42^, who found horizontal and vertical advection to be the primary drivers of vorticity in a heavy rainfall event west of Zhengzhou, China. In the Tehran case, the vortex was most pronounced around 850 hPa. While horizontal advection exhibited a long-lasting influence, vertical advection, divergence, and tilting became significant several hours prior to the flood. The substantial low-level humidity and convective heating promoted the formation of the MCV at lower altitudes.

The analysis highlights that all vorticity budget terms collectively influence MCV development, with vertical advection serving a compensatory role. In contrast to some studies in China, where vorticity advection terms were smaller than other contributions^1^, and to Knievel and Johnson^13^, who identified tilting as the dominant term, our case demonstrates the importance of multiple interacting mechanisms. Relative vorticity extended vertically throughout the atmospheric column, while convergence of absolute vorticity was confined to the lower troposphere (surface to ~ 700 hPa), where horizontal and vertical winds reached their maximum. This indicates that both synoptic-scale and mesoscale winds contributed to MCV activity, consistent with prior work^13^.

The results emphasize that relying solely on synoptic-scale systems for precipitation forecasting is insufficient. While upper-level ridges and mid-level troughs influence large-scale circulation, mesoscale systems such as MCVs can dominate rainfall formation. MCVs enhance low-level convergence and upward motion, sustaining the transport of warm, moist air and promoting deep convection and persistent precipitation. These mesoscale systems form rapidly, have short lifespans, and evolve through self-sustaining mechanisms influenced by middle-to-low-level steering winds. Consequently, understanding their characteristics and interactions is crucial for improving precipitation forecasts and issuing hazard warnings.

Finally, it is important to note that this study relied on the ERA5 reanalysis dataset to calculate vorticity budget terms. While temporal and spatial resolution and model schemes can influence the results, the qualitative conclusions regarding the relative importance and temporal evolution of vorticity tendencies are considered robust. ERA5 provides a dynamically consistent and observationally constrained dataset, suitable for examining the MCV dynamics during the study period.

## Data Availability

Meteorological variables were obtained from the European Centre for Medium-Range Weather Forecasts (ECMWF) via the Copernicus Climate Data Store (https://cds.climate.copernicus.eu/datasets/reanalysis-era5-pressure-levels?tab=download). Late-run Global Precipitation Measurement (GPM) satellite data were accessed from NASA’s GES DISC archive (https://gpm1.gesdisc.eosdis.nasa.gov/data/GPM_L3/GPM_3IMERGDL.07/). Infrared satellite images were obtained from EUMETSAT and accessed via Meteologix (https://meteologix.com/ir/satellite/top-alert-15min.html).
